# Carotenoids and Vitamin A in Breastmilk of Hong Kong Lactating Mothers and Their Relationships with Maternal Diet

**DOI:** 10.3390/nu14102031

**Published:** 2022-05-12

**Authors:** Zhou Lu, Yat-Tin Chan, Kenneth Ka-Hei Lo, Danyue Zhao, Vincy Wing-Si Wong, Yuk-Fan Ng, Wing-Wa Ho, Liz Sin Li, Hang-Wai Lee, Man-Sau Wong, Shi-Ying Li

**Affiliations:** 1 Department of Applied Biology and Chemical Technology, The Hong Kong Polytechnic University, Hong Kong 999077, China; lu.zh.lu@connect.polyu.hk (Z.L.); yat-tin-winson.chan@connect.polyu.hk (Y.-T.C.); kenneth.kh.lo@polyu.edu.hk (K.K.-H.L.); daisydy.zhao@polyu.edu.hk (D.Z.); wingsi_vincy@hotmail.com (V.W.-S.W.); fanny.ng@polyu.edu.hk (Y.-F.N.); wing-wa.ho@polyu.edu.hk (W.-W.H.); sin-liz.li@polyu.edu.hk (L.S.L.); alston.lee@polyu.edu.hk (H.-W.L.); 2 Centre for Eye and Vision Research (CEVR), 17W Hong Kong Science Park, Hong Kong 999077, China; 3 Research Institute for Future Food, The Hong Kong Polytechnic University, Hong Kong 999077, China

**Keywords:** carotenoids, vitamin A, breastmilk, maternal diets, dietary intakes, lactation, cross-sectional study

## Abstract

Carotenoids and vitamin A are nutrients crucial to infants’ development. To date, there is limited data on their availability in breastmilk and the associated dietary factors, especially in Hong Kong, where people follow a westernized Chinese diet. This study determined the selected breastmilk’s carotenoid and vitamin A (retinol) contents by ultraperformance liquid chromatography with photodiode detection (UPLC-PDA) and the dietary intakes by three-day food records in 87 Hong Kong lactating mothers, who were grouped into tertiles based on their daily carotenoid intake. Low vitamin A intake (530.2 ± 34.2 µg RAE/day) and breastmilk retinol level (1013.4 ± 36.8 nmol/L) were reported in our participants, suggesting a poor vitamin A status of the lactating participants having relatively higher socioeconomic status in Hong Kong. Mothers in the highest tertile (T3) had higher breastmilk carotenoid levels than those in the lowest (T1) (*p* < 0.05). There were significant associations between maternal carotenoid intakes and breastmilk lutein levels in the linear regression models (*p* < 0.05) regardless of dietary supplement intake. Furthermore, maternal dark green vegetable intakes were associated with breastmilk retinol, lutein, and β-carotene levels. These findings can serve as dietary references for lactating mothers to enhance breastmilk carotenoid and vitamin A contents for the benefits of child growth and development.

## 1. Introduction

Early life nutrition has an important role on child health and the risk of chronic disease in later stages of life [1]. Breastmilk is the sole food for exclusively breastfed infants for the first few months of life and breastmilk nutrition is associated with infant growth and development. It is well recognized that breastmilk provides infants with not only dietary energy, but also a variety of bioactive factors, such as immunoglobulins, stem cells, cytokines, hormones, carotenoids, and polyphenols [2,3] for further development and health.

Carotenoids are fat-soluble pigments mainly obtained from plants and play an important role in the brain, vision, and immune system development of children. As mammals are incapable of synthesizing carotenoids, carotenoids in humans are primarily obtained from the dietary intake of fruits, vegetables and some foods of animal sources (i.e., salmon, egg yolk) [4]. Approximately 50 carotenoids have been found in the human diet, mainly from plant origins [5]. Carotenoids can be classified into two groups according to their chemical structure, namely hydrocarbon carotenes (e.g., α-carotene, β-carotene, and lycopene) and their hydroxylated derivatives, xanthophylls (e.g., β-cryptoxanthin, lutein and zeaxanthin). Among them, α-carotene, β-carotene, and β-cryptoxanthin can be converted into vitamin A in the body and are therefore called pro-vitamin A carotenoids. Carotenoids have been found to have many biological properties such as antioxidant and anti-inflammatory properties and have demonstrated multiple beneficial effects on human health [5]. Furthermore, their beneficial effects on maternal and infant health are well documented in previous studies [5,6,7,8]. For example, lutein and zeaxanthin could protect infants’ retinal pigment epithelium from oxidative damage and retinopathy [6], lycopene is associated with a reduced rate of fetal growth restriction and respiratory distress syndrome [7], and β-carotene could prevent pregnancy complications (e.g., preeclampsia, preterm birth, and delivery of small-for-gestational-age babies [8]).

Vitamin A is a fat-soluble micronutrient present in three forms, including retinol, retinal, and retinoic acid. Retinol exists in the form of retinyl ester in some foods with animal sources, such as liver, eggs, dairy products, and oily fish; or in the form of provitamin A carotenoids as mentioned above. In practice, the intake of vitamin A is quantified in retinol activity equivalent (RAE), which is calculated as retinol + (β-carotene/12) + (α-carotene/24) + (β-cryptoxanthin/24) [9]. In the early stage of life, vitamin A is essential for gene expression, reproduction, growth and development, erythropoiesis, immune function, and vision [9]. Breastmilk vitamin A is critical to meet the needs of infants as well as to accumulate in liver stores needed after weaning [10]. Breastmilk from vitamin-A-depleted mothers may predispose their infants to the risk of vitamin A deficiency, which may trigger xerophthalmia, anemia and impaired iron metabolism, growth retardation, increased infectious morbidity, depressed immune response, diarrheal disease, respiratory tract infections, and increased risk of mortality [11].

Maternal diet has been found to have an influence on the nutritional composition of breastmilk [12] and subsequently impacts child health and growth. Hong Kong people generally adopt a westernized diet with high consumption of red meat, refined grains, or foods high in sugar and inadequate consumption of fruit and vegetables according to governmental dietary guidelines. It was reported from a recent study [13] conducted among 73 local lactating mothers that their daily fruit and vegetable intakes (68.3 and 119.3 g, respectively) could only attain about one third of the suggested value from the Department of Health in Hong Kong. Low intake of fruit and vegetables could affect carotenoid and vitamin A levels in breastmilk, which might hinder child development. To our knowledge, there is no local data about the carotenoid and retinol levels in the breastmilk of Hong Kong mothers. This study aims to determine the carotenoid and retinol levels in the breastmilk of Hong Kong lactating mothers and examine the associated dietary factors. Moreover, through associating dietary carotenoid intakes with breastmilk carotenoid levels, this study could provide some evidence on how diet may become one modifiable factor to enhance nutrient levels in breastmilk.

## 2. Materials and Methods

### 2.1. Justification of Sample Size

The optimum sample size of the current study was estimated based on a previous study [14], which had measured the breastmilk carotenoid contents of 178 Guangzhou lactating mothers (280.7 ± 50.7 nmol/L, estimated Mean ± SD) and had enrolled the most similar subjects to our study in terms of ethnicity, geography, and culture. Cochran’s formula was then applied to the above data to calculate the optimal sample size as illustrated by Wong et al. [13]: $n\geq {\left(\frac{ZS}{E}\right)}^{2}$, where *n* is the estimated sample size, *S* is the standard deviation, *Z* is the confidence level (95% confidence = 1.96), and *E* is the range of possible random error. Suppose that the possible random error (*E*) was 4–5% of the mean value, the optimal sample size (*n*) was estimated as $n\geq {\left(\frac{1.96\;\times \;50.7}{280.7\;\times \;4\%\;to\;280.7\;\times \;5\%}\right)}^{2}=50\;\mathrm{to}\;78.$

### 2.2. Subject Recruitment

**Figure 1:**
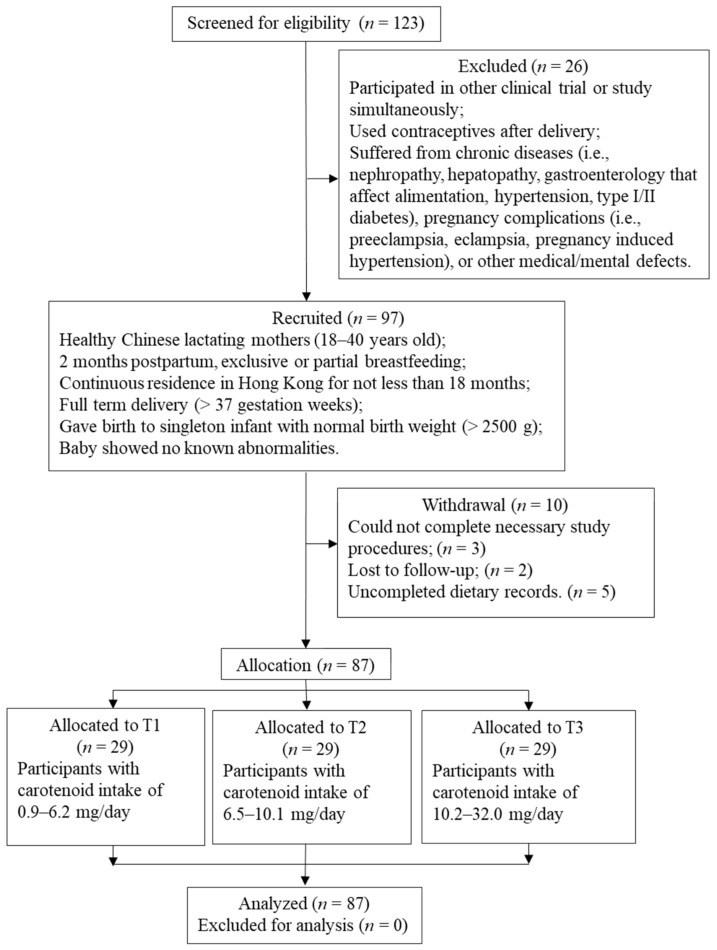
Subject recruitment flow chart of the study.

Lactating mothers were recruited via convenience sampling with the use of posters and email announcements as well as online social networking platforms between August 2014 and June 2015 (Figure 1). Eligibility of the subjects was determined according to the inclusion and exclusion criteria listed in Figure 1. This study included 87 eligible participants, and the demographic information of the participants are shown in detail in Table 1. Ethics approval was obtained from the Human Subjects Ethics Sub-Committee (reference number: HSEARS20150306002) and the biological safety and chemical safety of study were approved by the Health, Safety and Environment Office, The Hong Kong Polytechnic University, Hong Kong.

### 2.3. Breastmilk Sample Collection

Breastmilk samples were collected at The Hong Kong Polytechnic University from 87 eligible Hong Kong lactating women (Table 1). All samples were collected in the same time slot during summer to reduce the influence of diurnal and seasonal changes on breastmilk composition. On the day of collection, participants were required to empty the entire content of one breast using an electric breast pump (mini electric breast pump, MEDELA Inc., Mchenry, IL, USA) or hands. The samples were then aliquoted, coded, and stored at −80 °C until further analysis.

### 2.4. Chemicals and Standards

All chemicals were purchased from Sigma-Aldrich (St. Louis, MO, USA) with the highest purity available. Solvents were all of HPLC grade and were purchased from Anaqua (Cleveland, OH, USA). Water was double distilled and deionized via the Milli-Q system. All standards, including lutein, all-trans-retinol (Acros), β-carotene, lycopene (J&K, Beijing, China), and internal standard trans-β-Apo-8′-carotenal (Sigma-Aldrich, St. Louis, MO, USA) were of HPLC grade. All the carotenoids surveyed in this study are parental compounds.

### 2.5. Carotenoid and Retinol Extraction Method

The analytes were extracted from breastmilk according to a previously published method by Webb et al. [16] with modification. Briefly, to 1 mL of breastmilk, 750 μL of ethyl alcohol, 250 μL of an ethanolic solution of the internal standard (2.4 μmol/L trans-β-8-apo-carotenal), and 500 μL of a 20% L-ascorbic acid (*w*/*v*) aqueous solution were added. For saponification, the samples were kept at 50 °C for 15 min with agitation after the addition of 2 mL of 60% KOH (*w*/*v*) aqueous solution. Analytes were extracted from each sample three times using a total of 6 mL of n-hexane. To remove potassium hydroxide, the combined hexane fractions from each sample were washed with 1 mL of Milli-Q water and 750 μL of ethyl alcohol. The washed organic fractions were then evaporated to dry under nitrogen at 30 °C. The resulting residue of each sample was reconstituted by adding 100 μL of 1:1 ethyl alcohol/1,4-dioxane (*v*/*v*) and 150 μL of acetonitrile. After filtering with 0.22 μm polytetrafluoroethylene filters, the samples were analyzed by the UPLC system.

### 2.6. Carotenoid and Vitamin A Analysis by UPLC-PDA

The four analytes (lutein, β-carotene, lycopene, and retinol) and the internal standard were quantified using the Waters Acquity Ultra-Performance Liquid Chromatography system with a Photodiode-Array Detection (PDA) detector. Chromatographical separation was achieved with an Agilent Eclipse Plus C18 column (150 mm × 4.6 mm, 3.5 μm particle size) connected to a trident guard cartridge, and the flow rate was 1.1 mL/min. For the PDA eλ detector, a wavelength at 300 nm was selected for retinol, 445 nm for lutein and β-carotene, and 470 nm for lycopene. For all standard solutions and samples, the injection volume was 10 μL. The vitamin A level in breastmilk was determined by the retinol level in breastmilk. All data were acquired and processed using the Empower 3 software (Waters, Milford, MA, USA). Data quantifications were accomplished by internal standard calibration performed in breastmilk matrix, with the final concentrations presented in nmol/L.

### 2.7. Dietary Intake Analysis

The daily intakes of energy and selected nutrients were assessed using 3-day dietary records, which were collected as described in a previous published study [13]. The 3-day dietary records were noted for three consecutive days prior to the breastmilk sample collection date. The 3-day dietary record sheet was sent to the participants through e-mail after the breastmilk collection appointment had been confirmed. The time, types, and amounts of food and beverage consumed were recorded by the participants. In addition, condiments and oils consumed were estimated using the standard household measures. On the day of breastmilk sampling, the dietary records were collected by our research staff, and any unclear or missing food items were checked. An album containing pictures of commonly consumed local foods, guidelines on estimating food portions and representations of different standardized portions were provided to participants for a more accurate recording of dietary intake. Furthermore, participants were interviewed for their specific dietary habits (e.g., extra sauce addition, fat removal from meat, etc.) to minimize estimation bias. Food items listed on the records were then entered into the Food Processor Nutrition Analysis and Fitness software (version 10.13.1, ESHA Research Inc, Salem, MA, USA), which contained nutrient information from the Food Composition Database of the USDA, Agricultural Research Service (2015): the China Food Composition (Chinese Edition, 2002); and the Centre for Food Safety, Hong Kong. The daily intakes of dark green vegetables (i.e., broccoli, collard greens, dandelion greens, kale, lettuces, mustard greens, spinach, radish greens, watercress, turnip greens, swiss chard, etc.), light-colored vegetables (i.e., cauliflower, Chinese white cabbage, celery, baby Chinese cabbage, etc.), and red and orange vegetables (i.e., carrots, sweet potatoes, pumpkins, tomatoes, winter squash, red peppers, etc.) were also determined using the 3-day records. Daily intake was estimated by taking the average of the three days from the food sources and supplements (if any). In the present study, the daily intakes of energy, total fat, vitamin A (retinol activity equivalent), retinol, lutein + zeaxanthin, β-carotene, lycopene, α-carotene, β-cryptoxanthin, and total carotenoids were reported. The frequency, brand, name, and dosage of dietary supplements taken during lactation were self-reported by participants. Twenty-five participants reported taking vitamin-A-related supplements (i.e., Materna (contained 5400 μg of β-carotene and 462 μg RAE of vitamin A per serving)/Blackmore Pregnancy & Breastfeeding Pills (contained 4.8 mg of β-carotene per serving)/Centrum multivitamin (contained 600 μg of β-carotene, 1 mg of lutein, 600 μg of lycopene, and 300 μg RE of vitamin A per serving)/Nowfoods EVE Superior Women’s Multi (contained 3000 μg of β-carotene, 500 μg of lutein, and 500 μg of lycopene per serving)) 3–7 servings/week during lactation. This information was included in calculations of nutrient intakes.

### 2.8. Statistical Analysis

The results of the present study were expressed in the form of means, standard deviations, standard error of the mean, or 95% confidence intervals (CI). One-way ANOVA and chi-square test were used to detect subgroup differences of continuous and categorical variables, respectively.

In the present study, 87 eligible participants were first ranked and then divided into tertiles (T1, T2, T3) based on their total daily carotenoid intake obtained from 3-day dietary records. Participants in T3 (*n* = 29) had the highest level of carotenoid intake of 10.2–32.0 mg/day, T2 (*n* = 29) had the median level of carotenoid intake of 6.5–10.1 mg/day, and T1 (*n* = 29) had the lowest level of carotenoid intake of 0.9–6.2 mg/day. Sensitivity testing was performed by counting carotenoid intake from diets only (i.e., excluding the carotenoid intake from supplements), and then dividing the 87 participants into tertiles, with T3 (*n* = 29) consuming the most carotenoid (10.0–32.0 mg/day), T2 (*n* = 29) consuming the median carotenoid (4.7–9.3 mg/day), and T1 (*n* = 29) consuming the least carotenoid (0.9–4.5 mg/day). In this case, as 25 out of 87 participants reported to take vitamin-A-related supplements, the carotenoid intakes of these 25 participants would decrease (because the carotenoid intakes from supplements were not counted) while those of the 62 non-supplemented participants would remain the same. In addition, the relationship between types of vegetables (dark green vegetables, light-colored vegetables, red and orange vegetables) and breastmilk carotenoids was investigated among the 62 non-supplemented participants by dividing them into tertiles based on their vegetable intakes.

Moreover, the associations between maternal dietary intakes and breastmilk carotenoids were assessed by multivariable linear regression, adjusting for vitamin A intake, fat intake, educational level, age and BMI of participants, and the month of lactation. IBM^®^ SPSS Statistics 20 was used to perform all statistical analyses, with *p* < 0.05 (two-tailed) being considered as statistically significant.

## 3. Results

### 3.1. Participants’ Characteristics

The participants of the three groups had average ages of 32.3 years old and average BMI of 22.7 kg/m^2^. More than one third (37.9%) of the respondents were overweight or obese, and the carotenoid intake did not affect the maternal body weight status significantly. Vitamin A supplementation were significantly more prevalent among participants in Group T3 than T1 (41.4% vs. 10.3%), while other demographic features did not differ significantly by subgroup.

### 3.2. Dietary Characteristics of Hong Kong Lactating Mothers

The mean daily intake of vegetables, dark green vegetables, light-colored vegetables, red and orange vegetables, and fruit was 177.9 ± 10.5 g, 55.2 ± 5.4 g, 29.8 ± 4.8 g, 52.2 ± 6.1 g, and 100.4 ± 9.1 g, respectively (Table 2). The mean daily intake of vitamin A, retinol, lutein + zeaxanthin, β-carotene, lycopene, α-carotene, and β-cryptoxanthin was 530.2 ± 34.2 μg RAE, 327.9 ± 26.6 μg RE, 3.3 ± 0.4 mg, 3.5 ± 0.3 mg, 2.7 ± 0.4 mg, 332.9 ± 73.1 μg, and 104.9 ± 20.3 μg, respectively (Table 2). By dividing the 87 participants into tertiles based on their carotenoid intakes, significant differences among three groups were observed in total vegetable (*p* < 0.001), dark green vegetable (*p* = 0.03), and red and orange vegetable (*p* = 0.002) intakes. There were also significant differences in dietary vitamin A intakes among three groups (*p* = 0.004). Among the four nutrients being analyzed, the participants consumed β-carotene the most, followed by lutein + zeaxanthin, lycopene, and retinol. Significant intake differences (*p* < 0.05) were observed among three groups for lutein + zeaxanthin, β-carotene, lycopene, and α-carotene only.

The top five most-consumed food items for dark green vegetable, light-colored vegetable, red and orange vegetable, and fruit, and their carotenoid contents are listed in Supplementary Table S1. Dark greens, including choy sum, broccoli, Chinese kale, spinach, and water spinach, contributed the most to the lutein + zeaxanthin and β-carotene intakes of the participants. Red and orange vegetables, like carrots, tomatoes, red peppers, pumpkins, and sweet potatoes, are major food sources of β-carotene, with tomatoes also being the main source of lycopene. In addition, fruit and light-colored vegetables are noteworthy but less prominent sources of lutein + zeaxanthin and β-carotene intakes.

### 3.3. Carotenoid Levels in Breastmilk from Hong Kong Lactating Mothers

**Figure 2:**
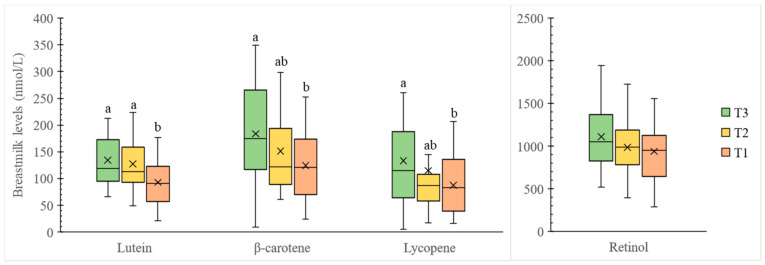
Box plots of major carotenoid and retinol contents (nmol/L) in breastmilk categorized by total carotenoid intake from food and supplements. Boxes represent the 25th, 50th, and 75th percentiles. Crosses represent mean. Whiskers represent either minimum/maximum or 25th/75th minus/plus 1.5 × interquartile range, whichever is closer to the median. Different superscript letters indicate a significant difference in carotenoid or retinol content in human milk from different groups (*p* < 0.05) based on one-way ANOVA test with post hoc Bonferroni test. Participants in T3 (*n* = 29) had the highest carotenoid intake of 10.2–32.0 mg/day, T2 (*n* = 29) had the median carotenoid intake of 6.5–10.1 mg/day, and T1 (*n* = 29) had the lowest carotenoid intake of 0.9–6.2 mg/day.

**Figure 3:**
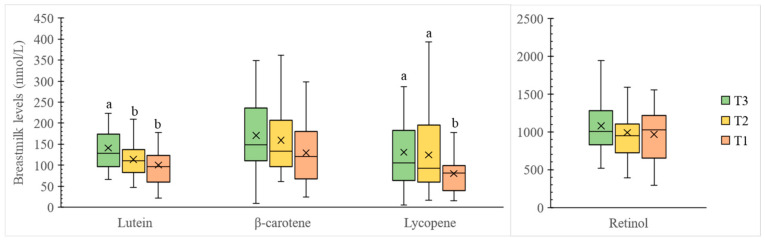
Box plots of major carotenoid and retinol contents (nmol/L) in breastmilk categorized by total carotenoid intake from food only. Boxes represent the 25th, 50th, and 75th percentiles. Crosses represent mean. Whiskers represent either minimum/maximum or 25th/75th minus/plus 1.5 × interquartile range, whichever is closer to the median. Different superscript letters indicate a significant difference in carotenoid or retinol content in human milk from different groups (*p* < 0.05) based on one-way ANOVA test with post hoc Bonferroni test. Participants in T3 (*n* = 29) had the highest carotenoid intake of 10.0–32.0 mg/day, T2 (*n* = 29) had the median carotenoid intake of 4.7–9.3 mg/day, and T1 (*n* = 29) had the lowest carotenoid intake of 0.9–4.5 mg/day.

The breastmilk of the 87 lactating mothers contains 118.2 ± 5.8 nmol/L lutein, 153.2 ± 8.7 nmol/L β-carotene, 111.9 ± 9.3 nmol/L lycopene, and 1013.4 ± 36.8 nmol/L retinol on average (Figure 2). Regarding the grouping by carotenoid intake, T3 mothers (with highest carotenoid intake) had significantly higher (*p* < 0.05) breastmilk lutein, β-carotene, and lycopene levels compared to T1 mothers (with lowest carotenoid intake), indicating positive associations between maternal dietary carotenoid intake and breastmilk carotenoid levels. After excluding the carotenoid intake from supplements, difference in breastmilk β-carotene concentrations among T1 to T3 was no longer significant (Figure 3, *p* > 0.05), while difference in breastmilk lutein and lycopene concentrations among T1 to T3 remained significant (Figure 3, *p* < 0.05).

### 3.4. Regression Analysis between Carotenoid Intake and Breastmilk Carotenoid Levels

We have conducted multivariable linear regression to evaluate the associations between maternal carotenoid intakes and breastmilk carotenoids (Table 3). In the fully adjusted model (Model 3), mothers with the highest carotenoid intake had significantly higher levels in breastmilk lutein (β = 45.1, 95% C.I. = 16.5, 73.7) and β-carotene (β = 63.0, 95% C.I. = 20.0, 106.1) compared to mothers with the lowest carotenoid intake. In the sensitivity analysis, carotenoid intake from dietary supplements was excluded and T1, T2, and T3 were regrouped based on dietary carotenoid intake (Table 4). The significant association between carotenoid intake and breastmilk lutein remained (β = 42.6, 95% C.I. = 14.4, 70.8), while the association between carotenoid intake and breastmilk lycopene became significant (β = 48.4, 95% C.I. = 2.0, 94.8).

Considering the food sources (i.e., different types of vegetables) of dietary carotenoids, dark green vegetables were positively associated with breastmilk retinol (Supplementary Table S2, β = 275.6, 95% C.I. = 82.2, 469.1), lutein (Supplementary Table S2, β = 54.3, 95% C.I. = 25.6, 83.1), and β-carotene (Supplementary Table S2, β = 92.5, 95% C.I. = 55.2, 129.8), while red and orange vegetables had no significant associations with breastmilk carotenoids after adjusting for confounders (Supplementary Table S3). Meanwhile, a higher consumption of light-colored vegetables was associated with elevated breastmilk lutein (Supplementary Table S4, β = 40.9, 95% C.I. = 9.2, 72.6).

## 4. Discussion

In the present study, levels of selected carotenoid and retinol were determined in the breastmilk from 87 lactating mothers, and the associations between dietary carotenoid intake and the respective concentrations in breastmilk were examined. There was a significant association between carotenoid intake and breastmilk lutein level regardless of dietary supplement intake. Furthermore, significant positive associations were found between dark green vegetable intake and breastmilk retinol, lutein, and β-carotene accumulation, as well as light-colored vegetable intake and breastmilk lutein level.

The modern Hong Kong diet is characterized as high consumption of red meat, refined grains, and foods high in sugar, with limited consumption of fruit and vegetables [13]. It was found in the present study that Hong Kong lactating women had a daily intake of fruit and vegetables (Table 2, 100.4, 177.9 g/day, respectively) below the dietary recommendation for Hong Kong population (3.0, 4.0–5.0 servings/day (i.e., 240, 320–400 g/day), respectively) [17]. Additionally, the intakes of total vegetables, dark green vegetables, and light-colored vegetables of our participants (Table 2, 177.9, 55.2, 29.8 g/day, respectively) were lower than those reported among Chinese populations (276.2, 90.8, 185.4 g/day, respectively) and Chinese lactating mothers (295.9, 94.7, 201.2 g/day, respectively) [18], which may reflect the relatively low vegetable intakes by Hong Kong lactating mothers in general. Although the average daily fruit intake of Hong Kong mothers (Table 2, 100.4 g/day) exceeded that of mothers from Mainland China (47.8 g/day) [18], 14.9% of the participants did not consume any fruit according to their 3-day dietary records.

Despite the low fruit and vegetable intake of our participants, the current intakes of lutein + zeaxanthin, β-carotene, and lycopene (Table 2) were comparable with those published previously [18,19]. The lutein + zeaxanthin and lycopene intakes of our participants (3.3, 2.7 mg/day) were lower than those reported in Polish mothers during the sixth month of lactation (3739.3, 7255.8 µg/day, respectively) [19], while the β-carotene intake was comparable (3514.5 vs. 3441.9 µg/day). Another study conducted among Shanghai’s lactating mothers, whose dietary habits should be quite similar to our participants, reported a comparable intake of lutein + zeaxanthin to the current study (3.3 vs. 3.3 mg/day) [18]. On the other hand, the participants’ mean dietary intake of vitamin A (530.2 ± 34.2 μg RAE/day) was around 60% lower than the RNI (1300 μg RAE/day) for lactating women according to the Chinese Dietary Reference Intakes (DRI) 2013 [20]. Among our participants, only 4 out of 87 achieved the RNI for vitamin A intake. Based on the data collected from the 3-day records, most commonly consumed food items rich in retinol by our participants are summarized in Supplementary Table S5. While 45.5 μg retinol was consumed from egg products daily on average, less retinol was consumed from dairy products (i.e., 1.0 μg/day from cheese, 33.4 μg/day from butter) that are also high in retinol. Although seafood is popular food in Hong Kong, only 2.8 μg/day, 2.8 μg/day, 0.3 μg/day, and 4.7 μg/day of retinol was consumed from salmon, mackerel, tuna, and prawn, respectively. Maternal vitamin A requirement increases during lactation to meet the increased needs imposed by the mammary gland, and vitamin A in breastmilk is essential to build up the infant’s liver stores, contributing to the prevention of vitamin A deficiency when lactation ends [21,22]. Therefore, lactating women are suggested to consume more retinol-rich food as mentioned above.

In this study, the breastmilk vitamin A (retinol) level observed among our participants was 1013.4 nmol/L, which was among the lowest levels in the world (world average = 1036.8~2880.1 nmol/L), though it is similar to those from the Chinese, Australian, and British populations (Table 5). In addition, the mean breastmilk vitamin A level of the 33 exclusive breastfeeding participants in the present study was 965.2 ± 57.1 nmol/L, which may not meet the infants’ vitamin A requirement (300 μg RAE/day, AI) as suggested by Chinese DRIs for 0–6 month infants [20] (assuming the intake of breastmilk is 750 mL/day for infants, the intake of vitamin A RAE is calculated as 965.2 nmol/L × 750 mL/day = 723.9 nmol/day = 207.4 μg/day). According to the World Health Organization, when retinol concentration in milk is below 1.05 μmol/L (1050 nmol/L), the infant’s body stores may be below the estimated critical amounts to supply for the increased requirements in the second half of childhood [23]. In the current study, around one third (36.4%) of lactating mothers in the first 6 months of lactation (*n* = 33) had milk retinol levels higher than 1.05 μmol/L. This finding is noteworthy, as vitamin A deficiency may occur in the children of exclusive breastfeeding mothers with low breastmilk vitamin A, which will hinder the infants’ health and development. A similar situation was reported among Chinese lactating mothers in a recent study [24], which indicated a high risk of vitamin A deficiency in Chinese mothers and infants, as a high percentage of Chinese populations (44%) had milk retinol level less than 1.05 µmol/L. Vitamin A is essential for newborn development, epithelia function and protection against infections. Vitamin A deficiency affects millions of preschool-age children, especially in developing countries. Inadequate intakes of vitamin A may lead to vitamin A deficiency, which can cause visual impairment in the form of night blindness and may increase the risk of illness and death from childhood infections. Interestingly, although breastmilk vitamin A levels of Chinese and Hong Kong lactating mothers are among the lowest around the world, vitamin A deficiency in Chinese children (9.3%) is not as prevalent as other developing countries [23]. Also, the prevalence of night blindness in Chinese preschool children (0.1%) is the lowest [23]. More studies should be done to explore the impacts of low breastmilk vitamin A levels on infants’ development and visual health in Hong Kong population.

In contrast, levels of carotenoids in the breastmilk of Hong Kong mothers are comparable to the world average level. Previous studies have shown that lutein and zeaxanthin, lycopene, α-carotene, β-carotene, and β-cryptoxanthin are the main carotenoids in human milk [14,25,26,29,30]. In the present study, β-carotene (153.2 ± 8.7 nmol/L) was found to be the most abundant carotenoid in the breastmilk from participants. Studies among other populations have found lower β-carotene levels, from 16.0 to 78.2 nmol/L, in mature milk (Table 5). Our study also showed that average lycopene concentration in breastmilk from participants was 111.9 nmol/L, while most studies from other populations have observed lower lycopene levels of 14.0 to 59.8 nmol/L in mature human milk (Table 5). However, a more recent study conducted in Poland [19] reported lycopene levels in breastmilk similar to our result. Regarding lutein, some studies have reported lower breastmilk lutein levels at 6.0–107.6 nmol/L compared to our finding (118.2 nmol/L), mainly among western populations, while some studies from China [14,18,26] reported similar or higher concentrations at 121.3–179.3 nmol/L. Differences in the breastmilk carotenoid contents in our study when compared to those reported by others could be attributed by the differences in dietary habits across populations, including food preference and food preparation methods, as well as breastmilk sampling methods [31]. In Hong Kong, vegetables are more often eaten cooked, with stir-frying, boiling, and steaming being three of the most prevalent cooking methods. Previous studies [32] have demonstrated that heat treatment of vegetables is able to raise the bioavailability or extractable amounts of carotenoids from vegetables such as tomatoes, carrots, and spinach, as it may break up the interactions between carotenoids and proteins in the vegetables and unstiffen the fibrous plant tissue [33]. Additionally, enzymes that may potentially oxidize carotenoids can be denatured by heat. On the other hand, the fat consumed with the stir-fried vegetables may aid carotenoid absorption [34]. Additionally, genetic frequency of homozygous rs6420424 A allele and homozygous rs11645428 G allele that are able to reduce the β-carotene conversion efficiency is significantly higher in Asian population, leading to an overall higher β-carotene level in Asian ethnic groups [35].

From the regression analysis, carotenoid intake was positively associated with the levels of breastmilk lutein and β-carotene (Table 3). This observation indicated an association between maternal carotenoid intake and breastmilk carotenoid level, which was consistent with previous evidence showing that dietary carotenoid intakes correlated positively with the carotenoid concentrations in breastmilk [19,36]. Some intervention studies have further ascertained such associations by reporting significantly increased carotenoid concentrations in breastmilk after being supplemented with lutein [37], red palm oil [38], Chlorella [39], carrots and tomato paste [40]. However, a recent study in Chinese lactating mothers [18] found no significant correlation between lutein and zeaxanthin intakes and their breastmilk levels. Similarly, another study conducted in Brazil [28] did not observe any associations between provitamin A intakes and β-carotene and retinol levels in breastmilk. Such discrepancies may be due to different dietary patterns among different populations, in which different bioavailability of carotenoids from different food sources could contribute to the difference in the availability of carotenoids in breastmilk [41].

It was noted that the association between carotenoid intake and breastmilk β-carotene was no longer significant after excluding the participants who had vitamin-A-related supplement intake (Table 4). This indicated the significant influence of supplements on breastmilk β-carotene. At present, the effects of different forms of vitamin A intervention on maternal breastmilk carotenoid concentrations are inconsistent. Canfield et al. [25] previously showed that maternal β-carotene supplementation elevated maternal breastmilk β-carotene concentrations and increased infant serum retinol significantly (*p* < 0.001). On the other hand, Gurgel et al. [42] pointed out that the effect of β-carotene supplements is conditioned to the maternal vitamin A status as the regulatory mechanism tends to maintain homeostasis [43]. In the present study, the significantly enhanced breastmilk β-carotene level after vitamin A supplementation corresponded to the low vitamin A status in Hong Kong lactating mothers.

As for dietary vegetable sources, dark green vegetable intake was significantly associated with breastmilk retinol, lutein, and β-carotene content (Supplementary Table S2, *p* < 0.05) in Hong Kong lactating mothers. On the other hand, maternal intake of red and orange vegetables did not show significant associations with breastmilk carotenoids in the fully adjusted model (Supplementary Table S3, Model 3). Meanwhile, light-colored vegetable intake was found to contribute to elevated breastmilk lutein levels significantly (Supplementary Table S4, *p* < 0.05). These findings were consistent with several multinational studies, which indicated that breastmilk carotenoid pattern was determined by the consumption of fruit and vegetables, especially the dark green ones [25,26]. A more recent study also pointed out the determinant role of dark green leafy vegetable consumption on the vitamin A status of women in Chamwino [44]. As a result, it is suggested that breastmilk retinol, lutein, and β-carotene levels can be significantly increased through a dietary pattern rich in vegetables during lactation. Among them, dark green vegetables are good sources of lutein and β-carotene (Supplementary Table S2). Recent studies indicated the important roles of carotenoids and retinol during infancy [5,6,8]. Based on the findings in the current study, lactating women are highly recommended to consume more vegetables, especially dark green vegetables [18,35], to increase the breastmilk carotenoid and vitamin A levels for supporting optimal infant development. In view of the lipid-soluble property of these micronutrients, it is also recommended to cook carotenoid-rich vegetables with oil or consume those vegetables together with fat-containing food (e.g., meat, oily fish, avocado) to facilitate intestinal absorption. Furthermore, lactating mothers are recommended to consume foods rich in vitamin A (retinol) such as eggs and vitamin-A-fortified dairy products as well as oily fish (twice a week), which are safe ways to improve the vitamin A status in lactating mothers and support infant health.

To the best of our knowledge, the present study is the first to quantify breastmilk levels of vitamin A (retinol) in addition to the commonly consumed carotenoids in Hong Kong lactating mothers. In addition, maternal dietary carotenoid intakes were estimated to assess their associations with breastmilk carotenoid levels. By exploring the associated dietary factors on breastmilk carotenoids, our study may provide some scientific evidence for the development of suitable health measures to improve the well-being of breastfed infants and lactating mothers in Hong Kong. More importantly, our study plays an important role in providing dietary recommendation to local lactating mothers on fruit and vegetable intakes. However, it is acknowledged that interpretation of the present data has some limitations. Firstly, the recruited participants generally had a higher socioeconomic status and educational level above the municipal average, which may result in selection bias in the present study. Secondly, the cross-sectional design of the current study may not be sufficient to provide evidence of a relationship between dietary exposure and breastmilk carotenoid levels. Thirdly, maternal serum was not collected in this study, so the associations between breastmilk carotenoid levels and plasma carotenoid levels cannot be constructed. Fourthly, as the HPLC peaks of lutein and zeaxanthin may appear at a similar retention time, the lutein level reported in this study may be overestimated. Fifthly, the intake of energy and selected nutrients may be underestimated, as they were calculated from the 3-day dietary records only. Finally, some other dietary carotenoids (e.g., zeaxanthin, β-cryptoxanthin) should be determined in further studies to establish a more complete breastmilk carotenoid database in Hong Kong lactating mothers. Despite the limitations mentioned above, preliminary data of dietary influence on breastmilk carotenoid levels have been provided in the current study, which may lay a foundation for further interventional study design.

## 5. Conclusions

The concentrations of breastmilk lutein, β-carotene, lycopene, and retinol of 87 healthy lactating women from Hong Kong, along with their diets, were examined in the present study. This study indicated significant influences of maternal carotenoid intakes on breastmilk carotenoids levels, among which carotenoid intake from supplements mainly affects breastmilk β-carotene, while carotenoid intake from non-supplement food sources mainly affects breastmilk lutein and lycopene. Moreover, maternal dark green vegetable and light-colored vegetable intakes were found to have significant positive associations with breastmilk retinol, lutein, and β-carotene levels, which implied that breastmilk vitamin A and carotenoids could be increased through a vegetable-rich diet. In addition, vitamin-A-fortified food is also recommended to lactating mothers to increase breastmilk vitamin A levels. The findings from the present study may serve as evidence for lactating mothers to optimize their breastmilk carotenoid contents for the benefit of child growth and development.

## Figures and Tables

**Table 1:** Anthropometric and socioeconomic characteristics of 87 Hong Kong lactating mothers.

Variables	T1 (*n* = 29)	T2 (*n* = 29)	T3 (*n* = 29)	Total (*n* = 87)	*p*-Value
Age (years)	31.8 ± 4.0	32.0 ± 3.1	33.2 ± 3.8	32.3 ± 3.6	0.30
Weight (kg)	56.7 ± 8.5	59.8 ± 10.1	57.2 ± 7.6	57.9 ± 8.8	0.35
Height (m)	1.60 ± 0.05	1.61 ± 0.06	1.58 ± 0.05	1.60 ± 0.05	0.21
BMI (kg/m^2^)	22.2 ± 3.1	23.2 ± 3.9	22.9 ± 3.3	22.7 ± 3.4	0.54
Body weight status ^I^ (%)					0.47
Underweight	0 (0.0%)	2 (6.9%)	1 (3.5%)	3 (3.5%)	
Normal weight	20 (69.0%)	16 (55.2%)	15 (51.7%)	51 (58.6%)	
Overweight or obese	9 (31.0%)	11 (37.9%)	13 (44.8%)	33 (37.9%)	
Vitamin A-related supplemented (%)	3 (10.3%) ^aII^	10 (34.5%) ^b^	12 (41.4%) ^b^	25 (28.7%)	0.02
Education ^III^ (%)					0.15
High	20 (69.0%)	22 (75.9%)	26 (89.7%)	68 (78.2%)	
Low	9 (31.0%)	7 (24.1%)	3 (10.3%)	19 (21.8%)	
Monthly family income ^I^^V^ (%)					0.10
High	17 (58.6%)	18 (62.1%)	24 (82.8%)	59 (67.8%)	
Low	12 (41.4%)	11 (37.9%)	5 (17.2%)	28 (32.2%)	
Occupation (%)					0.93
Housewife	6 (20.7%)	6 (20.7%)	5 (17.2%)	17 (19.5%)	
Full-time work	23 (79.3%)	23 (79.3%)	24 (82.8%)	70 (80.5%)	
Lactation stage (%)					0.47
2–6 months	12 (41.4%)	9 (31.0%)	12 (41.4%)	33 (37.9%)	
6–12 months	6 (20.7%)	12 (41.4%)	10 (34.5%)	28 (32.2%)	
12–24 months	11 (37.9%)	8 (27.6%)	7 (24.1%)	26 (29.9%)	
Infant gender (%)					0.40
Male	15 (51.7%)	20 (69.0%)	18 (62.1%)	53 (60.9%)	
Female	14 (48.3%)	9 (31.0%)	11 (37.9%)	34 (39.1%)	
Birth weight (kg)	3.17 ± 1.15	3.13 ± 0.32	3.08 ± 0.29	3.13 ± 0.70	0.90

Data are expressed as mean ± SD/number of participants (percentages). ^I^ Underweight: BMI < 18.5; Normal weight: BMI 18.5–22.9; Overweight: BMI 23.0–24.9; Obese: BMI ≥ 25.0; ^II^ Different superscript letters indicate a significant difference in characteristics from different groups (*p* < 0.05); ^III^ High education: tertiary education or above; ^IV^ High monthly family income: income > HKD 30,000 [15].

**Table 2:** Energy intake, intakes of fruit and vegetables, and selected nutrients of the 87 Hong Kong lactating women.

Energy, Food Group, and Nutrients (Unit)	T1 (*n* = 29)	T2 (*n* = 29)	T3 (*n* = 29)	Total (*n* = 87)	*p*-Value ^I^
Vegetables (g/day)	116.8 ± 10.1 ^bII^	192.4 ± 19.0 ^a^	224.5 ± 19.6 ^a^	177.9 ± 10.5	*p* < 0.001
Dark green vegetables (g/day)	35.3 ± 5.2 ^b^	60.4 ± 10.7 ^ab^	69.8 ± 11.2 ^a^	55.2 ± 5.4	0.03
Light-colored vegetables (g/day)	20.0 ± 5.8	30.1 ± 7.3	39.3 ± 11.5	29.8 ± 4.8	0.26
Red and orange vegetables (g/day)	26.8 ± 6.3 ^b^	52.4 ± 10.5 ^ab^	77.3 ± 12.9 ^a^	52.2 ± 6.1	0.002
Fruit (g/day)	78.8 ± 15.5 ^b^	98.4 ± 13.3 ^ab^	123.8 ± 19.2 ^a^	100.4 ± 9.1	0.13
Energy (kcal/day)	2306.2 ± 82.0 ^b^	2343.6 ± 71.1 ^ab^	2529.6 ± 67.0 ^a^	2393.1 ± 41.9	0.06
Fat (g/day)	96.5 ± 5.1	96.4 ± 4.0	105.6 ± 4.2	99.5 ± 2.5	0.23
Vitamin A (μg RAE/day)	399.4 ± 50.2 ^b^	520.0 ± 62.0 ^ab^	671.2 ± 61.4 ^a^	530.2 ± 34.2	0.004
Retinol (μg RE/day)	325.0 ± 51.8	361.2 ± 58.0	297.4 ± 29.7	327.9 ± 26.6	0.62
Lutein + zeaxanthin (mg/day)	1.4 ± 0.1 ^b^	2.4 ± 0.2 ^b^	6.2 ± 1.0 ^a^	3.3 ± 0.4	*p* < 0.001
β-Carotene (mg/day)	1.2 ± 0.2 ^c^	3.1 ± 0.4 ^b^	6.2 ± 0.5 ^a^	3.5 ± 0.3	*p* < 0.001
Lycopene (mg/day)	0.9 ± 0.2 ^b^	2.4 ± 0.4 ^b^	4.9 ± 0.9 ^a^	2.7 ± 0.4	*p* < 0.001
α-Carotene (μg/day)	43.5 ± 20.9 ^b^	277.0 ± 81.9 ^b^	678.1 ± 194.4 ^a^	332.9 ± 73.1	0.001
β-Cryptoxanthin (μg/day)	75.5 ± 30.6	97.1 ± 34.3	142.0 ± 43.3	104.9 ± 20.3	0.40
Total carotenoids (mg RE/day)	3.6 ± 0.3 ^c^	8.3 ± 0.2 ^b^	18.2 ± 1.2 ^a^	10.0 ± 0.8	*p* < 0.001

Data are calculated from the 3-day dietary records and expressed as mean ± SEM. ^I^ *P*-value obtained from one-way ANOVA test with post hoc Bonferroni test; ^II^ different superscript letters indicate a significant difference in intakes from different groups (*p* < 0.05).

**Table 3:** Regression coefficients on the association between carotenoid intake and the levels of breastmilk retinol and carotenoids.

Breastmilk Retinol and Carotenoids	Categories in Carotenoid Intake	Model 1 (95% C.I.) ^I^	Model 2 (95% C.I.) ^I^	Model 3 (95% C.I.) ^I^
Retinol	T3	172.1 (−5.3, 349.5)	153.4 (−33.0, 339.8)	113.9 (−83.3, 311.1)
	T2	46.4 (−131.0, 223.7)	38.9 (−142.9, 220.5)	20.9 (−163.2, 205.0)
	T1	Ref	Ref	Ref
Lutein	T3	41.8 (15.2, 68.4) **^II^	45.9 (19.1, 72.7) **	45.1 (16.5, 73.7) **
	T2	34.7 (8.1, 61.3) *	38.5 (12.4, 64.6) **	37.5 (10.9, 64.2) **
	T1	Ref	Ref	Ref
β-Carotene	T3	59.5 (18.8, 100.2) **	62.8 (22.5, 103.0) **	63.0 (20.0, 106.1) **
	T2	26.6 (−14.1, 67.3)	33.6 (−5.6, 72.9)	33.1 (−7.0, 73.3)
	T1	Ref	Ref	Ref
Lycopene	T3	45.5 (0.9, 90.1) *	38.8 (−6.7, 84.3)	38.1 (−10.6, 86.7)
	T2	27.1 (−17.5, 71.7)	27.5 (−16.9, 71.8)	27.3 (−18.1, 72.7)
	T1	Ref	Ref	Ref

^I^ Model 1: Univariable analysis; Model 2: Multivariable analysis adjusted for maternal age, BMI, education, and lactation stage; Model 3: Multivariable analysis adjusted for maternal age, BMI, education, lactation stage, fat, and vitamin A intake; ^II^ * *p* < 0.05, ** *p* < 0.01.

**Table 4:** Regression coefficients on the association between carotenoid intake and the levels of breastmilk retinol and carotenoids after excluding supplemental carotenoid intake.

Breastmilk Retinol and Carotenoids	Categories in Carotenoid Intake	Model 1 (95% C.I.) ^I^	Model 2 (95% C.I.) ^I^	Model 3 (95% C.I.) ^I^
Retinol	T3	111.2 (−68.5, 291.0)	97.5 (−86.7, 281.8)	54.1 (−139.1, 247.2)
	T2	23.7 (−156.0, 203.5)	13.9 (−169.6, 197.4)	−3.7 (−189.7, 182.4)
	T1	Ref	Ref	Ref
Lutein	T3	40.6 (13.7, 67.5) **^II^	43.7 (17.1, 70.4) **	42.6 (14.4, 70.8) **
	T2	13.6 (−13.2, 40.5)	17.8 (−8.7, 44.3)	16.1 (−11.1, 43.2)
	T1	Ref	Ref	Ref
β-Carotene	T3	41.3 (−0.4, 82.9)	43.4 (2.9, 83.9) *	40.9 (−2.1, 83.9)
	T2	29.6 (−12.0, 71.3)	32.8 (−7.5, 73.1)	31.5 (−9.9, 72.9)
	T1	Ref	Ref	Ref
Lycopene	T3	51.2 (7.2, 95.2) *	48.6 (5.0, 92.3) *	48.4 (2.0, 94.8) *
	T2	44.8 (0.7, 88.8) *	47.1 (3.6, 90.6) *	48.1 (3.4, 92.7) *
	T1	Ref	Ref	Ref

^I^ Model 1: Univariable analysis; Model 2: Multivariable analysis adjusted for maternal age, BMI, education, and lactation stage; Model 3: Multivariable analysis adjusted for maternal age, BMI, education, lactation stage, fat, and vitamin A intake; ^II^ * *p* < 0.05, ** *p* < 0.01.

**Table 5:** Comparison of carotenoid and retinol levels in breastmilk with other populations.

	Lutein (nmol/L)	β-Carotene (nmol/L)	Lycopene (nmol/L)	Retinol (nmol/L)	References
Hong Kong	118.2 ± 5.8	153.2 ± 8.7	111.9 ± 9.3	1013.4 ± 36.8	Current study
Australia	27.0 ± 2.0	60.0 ± 7.0	31.0 ± 2.0	1086.0 ± 55.0	[25]
Canada	30.0 ± 1.0	36.0 ± 3.0	30.0 ± 2.0	1188.0 ± 66.0	
Chile	57.0 ± 5.0	44.0 ± 4.0	21.0 ± 2.0	1242.0 ± 85.0	
China	76.0 ± 8.0	48.0 ± 4.0	14.0 ± 1.0	1043.0 ± 88.0	
Japan	77.0 ± 4.0	62.0 ± 5.0	23.0 ± 2.0	1230.0 ± 63.0	
Mexico	44.0 ± 3.0	51.0 ± 5.0	32.0 ± 2.0	1321.0 ± 87.0	
Philippines	35.0 ± 3.0	22.0 ± 2.0	16.0 ± 2.0	1624.0 ± 94.0	
UK	27.0 ± 2.0	48.0 ± 3.0	34.0 ± 2.0	1052.0 ± 50.0	
USA	26.0 ± 1.0	37.0 ± 4.0	22.0 ± 2.0	1227.0 ± 87.0	
China	180.0	75.0	17.5	-	[26]
Mexico	80.0	37.5	40.0	-	
USA	70.0	70.0	57.5	-	
China	179.3 ± 14.4	-	-	-	[18]
Brazil	25.0 ± 3.0	16.0 ± 2.0	16.0 ± 3.0	620.0 ± 50.0	[27]
Brazil	6.0 ± 1.0	18.0 ± 2.0	-	1400.0 ± 100.0	[28]
USA	27.3 ± 16.4	36.1 ± 17.1	18.8 ± 9.5	1152.0 ± 488.7	[29]
Beijing	52.7 ± 3.3	31.7 ± 2.1	31.7 ± 1.7	-	[14]
Suzhou	105.5 ± 5.6	44.7 ± 2.9	31.7 ± 1.6	-	
Guangzhou	121.3 ± 7.0	50.3 ± 3.7	39.1 ± 2.7	-	
German	107.6 ± 48.0	78.2 ± 46.2	59.8 ± 38.9	2900.0 ± 1120.0	[30]
Poland	37.1	33.3	110.1	-	[19]

Data are expressed as mean ± SEM or mean.

## Data Availability

The data presented in this study are available on request from the corresponding authors.

## Supplementary Materials

The following supporting information can be downloaded at: https://www.mdpi.com/article/10.3390/nu14102031/s1, Table S1: Daily intake of carotenoid from the most frequently consumed dark green vegetables, light-colored vegetables, red and orange vegetables, and fruits; Table S2: Regression coefficients on the association between dark leafy vegetable intake and the levels of breastmilk carotenoids and retinol among the 62 participants without supplement intake; Table S3: Regression coefficients on the association between red and orange vegetable intake and the levels of breastmilk carotenoids among the 62 participants without supplement intake; Table S4: Regression coefficients on the association between light leafy vegetable intake and the levels of breastmilk carotenoids among the 62 participants without supplement intake; Table S5: Daily intake of retinol from the most frequently consumed food items rich in retinol.
