# Supplementary material for: Carotenoids and Vitamin A in Breastmilk of Hong Kong Lactating Mothers and Their Relationships with Maternal Diet
Source: Nutrients. 2022 May 12;14(10):2031. doi: 10.3390/nu14102031 (PMC9148123; doi:10.3390/nu14102031)
Supplement: Supplementary file 1 [file nutrients-14-02031-s001.zip › nutrients-1666486-supplementary.pdf]

Supplementary Table S1. Daily intake of carotenoid from the most frequently consumed dark green vegetables, light-colored vegetables, red and orange vegetables, and fruits.

Food group	Food item	Carotenoid content (μg/100g)			Mean carotenoid consumption (μg/day)[I]		
		Lutein	β-Carotene	Lycopene	Lutein	β-Carotene	Lycopene
Dark green vegetable	Choy sum	2848.1	2012.9	0.0	599.3	423.6	0.0
	Broccoli	1080.0	929.0	0.0	84.8	73.0	0.0
	Chinese kale	912.0	983.0	0.0	34.6	37.3	0.0
	Spinach	11308.0	6288.0	0.0	140.8	78.3	0.0
	Water spinach	8928.5	5539.0	0.0	465.2	288.6	0.0
Light-colored vegetable	Chinese white cabbage	1254.5	30.0	0.0	66.8	1.6	0.0
	Celery	329.0	313.0	0.0	4.9	4.6	0.0
	Shredded cabbage	30.0	42.0	0.0	1.5	2.1	0.0
	Baby Chinese cabbage	1254.5	202.6	0.0	10.1	1.6	0.0
	Cauliflower	29.0	7.0	0.0	0.3	0.1	0.0
Red and orange vegetable	Tomato	94.0	293.0	3041.0	11.0	34.3	355.4
	Carrot	687.0	8332.0	0.0	40.4	489.7	0.0
	Red pepper	47.0	1525.0	0.0	0.3	9.9	0.0
	Pumpkin	1014.0	2096.0	0.0	22.2	45.9	0.0
	Sweet potato	0.0	11509.0	0.0	0.0	273.4	0.0
Fruit	Orange	129.0	87.0	0.0	19.7	13.3	0.0
	Apple	29.0	27.0	0.0	3.7	3.4	0.0
	Banana	22.0	26.0	0.0	1.6	1.9	0.0
	Pear	44.0	14.0	0.0	1.3	0.4	0.0
	Pineapple	0.0	35.0	0.0	0.0	0.7	0.0

[I]Mean carotenoid consumption refers to the daily intake of carotenoid from a specific food item by the participants (n = 87) on average.

Supplementary Table S2. Regression coefficients on the association between dark leafy vegetable intake and the levels of breastmilk carotenoids and retinol among the 62 participants without supplement intake.

Breastmilk retinol and carotenoids	Categories in carotenoid intake	Model 1 (95% C.I.) [I]	Model 2 (95% C.I.) [I]	Model 3 (95% C.I.) [I]
Retinol	T3	268.9 (80.5, 457.3)**[II]	280.2 (89.5, 470.9)**	275.6 (82.2, 469.1)**
	T2	144.0 (-44.4, 332.4)	144.3 (-49.2, 337.8)	155.8 (-42.0, 353.7)
	T1	Ref	Ref	Ref
Lutein	T3	56.2 (26.9, 85.5)***	56.2 (27.1, 85.3)***	54.3 (25.6, 83.1)***
	T2	40.4 (11.1, 69.7)**	36.2 (6.7, 65.7)*	39.1 (9.8, 68.5)*
	T1	Ref	Ref	Ref
β-Carotene	T3	95.4 (55.1, 135.7)***	95.1 (56.9, 133.3)***	92.5 (55.2, 129.8)***
	T2	56.3 (16.0, 96.6)**	47.3 (8.5, 86.1)*	52.6 (14.5, 90.8)*
	T1	Ref	Ref	Ref
Lycopene	T3	21.4 (-39.9, 82.8)	21.3 (-38.1, 80.6)	20.5 (-40.1, 81.1)
	T2	4.1 (-57.2, 65.5)	-11.4 (-71.7, 48.8)	-11.0 (-72.9, 51.0)
	T1	Ref	Ref	Ref

[I]Model 1: Univariable analysis; Model 2: Multivariable analysis adjusted for maternal age, BMI, education, and lactation stage; Model 3: Multivariable analysis adjusted for maternal age, BMI, education, lactation stage, fat, and vitamin A intake; [II]* $p < 0.05$, ** $p < 0.01$.

Supplementary Table S3. Regression coefficients on the association between red and orange vegetable intake and the levels of breastmilk carotenoids among the 62 participants without supplement intake.

Breastmilk retinol and carotenoids	Categories in carotenoid intake	Model 1 (95% C.I.) [I]	Model 2 (95% C.I.) [I]	Model 3 (95% C.I.) [I]
Retinol	T3	-63.7 (-257.8, 130.3)	-51.9 (-254.7, 150.8)	-48.9 (-254.3, 156.5)
	T2	131.8 (-62.2, 325.9)	158.4 (-59.0, 375.7)	177.3 (-43.2, 397.8)
	T1	Ref	Ref	Ref
Lutein	T3	34.3 (2.7, 65.8)*[II]	28.5 (-3.8, 60.7)	29.0 (-3.2, 61.3)
	T2	6.2 (-25.3, 37.7)	-5.5 (-40.1, 29.1)	-1.4 (-36.0, 33.3)
	T1	Ref	Ref	Ref
β-Carotene	T3	13.3 (-33.9, 60.4)	-1.3 (-48.1, 45.6)	0.3 (-46.2, 46.7)
	T2	-4.1 (-51.2, 43.1)	-19.2 (-69.5, 31.0)	-12.3 (-62.2, 37.5)
	T1	Ref	Ref	Ref
Lycopene	T3	27.3 (-33.8, 88.5)	17.3 (-43.8, 78.4)	17.2 (-45.5, 79.9)
	T2	23.6 (-37.5, 84.8)	-9.3 (-74.8, 56.2)	-7.4 (-74.7, 59.9)
	T1	Ref	Ref	Ref

[I]Model 1: Univariable analysis; Model 2: Multivariable analysis adjusted for maternal age, BMI, education, and lactation stage; Model 3: Multivariable analysis adjusted for maternal age, BMI, education, lactation stage, fat, and vitamin A intake; [II]* $p < 0.05$, ** $p < 0.01$.

Supplementary Table S4. Regression coefficients on the association between light leafy vegetable intake and the levels of breastmilk carotenoids among the 62 participants without supplement intake.

Breastmilk retinol and carotenoids	Categories in carotenoid intake	Model 1 (95% C.I.) [I]	Model 2 (95% C.I.) [I]	Model 3 (95% C.I.) [I]
Retinol	T3	158.9 (-37.8, 355.5)	147.6 (-60.0, 355.1)	139.8 (-71.5, 351.1)
	T2	100.8 (-95.8, 297.4)	99.3 (-103.3, 302.0)	93.2 (-119.7, 306.0)
	T1	Ref	Ref	Ref
Lutein	T3	33.2 (1.5, 64.9)*[II]	43.5 (11.7, 75.2)**	40.9 (9.2, 72.6)*
	T2	23.5 (-8.2, 55.2)	26.0 (-5.0, 57.0)	21.1 (-10.8, 53.1)
	T1	Ref	Ref	Ref
β-Carotene	T3	-19.1 (-65.8, 27.7)	-6.5 (-53.7, 40.7)	-11.1 (-57.6, 35.4)
	T2	10.3 (-36.4, 57.1)	12.9 (-33.2, 59.0)	7.1 (-39.7, 53.9)
	T1	Ref	Ref	Ref
Lycopene	T3	-23.4 (-84.3, 37.6)	-20.1 (-81.5, 41.4)	-22.1 (-84.7, 40.5)
	T2	-34.5 (-95.5, 26.5)	-26.9 (-86.8, 33.1)	-32.6 (-95.6, 30.5)
	T1	Ref	Ref	Ref

[I]Model 1: Univariable analysis; Model 2: Multivariable analysis adjusted for maternal age, BMI, education, and lactation stage; Model 3: Multivariable analysis adjusted for maternal age, BMI, education, lactation stage, fat, and vitamin A intake; [II]* $p < 0.05$, ** $p < 0.01$.

Supplementary Table S5. Daily intake of retinol from the most frequently consumed food items rich in retinol.

Food group	Food item	Retinol content (μg/100g)	Mean retinol intake (μg/day)[I]
Egg	Egg	148.0	45.5
Seafood	Salmon	69.0	2.8
	Mackerel	54.0	2.8
	Tuna	17.0	0.3
	Prawn	90.0	4.7
Dairy product	Cheese	196.0	1.0
	Butter	671.0	33.4

[I]Mean retinol intake refers to the daily intake of retinol from a specific food item by the participants (n = 87) on average.
